# An Efficient and Scalable Method for the Production of Immunogenic SARS-CoV-2 Virus-like Particles (VLP) from a Mammalian Suspension Cell Line

**DOI:** 10.3390/vaccines11091469

**Published:** 2023-09-09

**Authors:** Stefan Hirschberg, Fatemeh Ghazaani, Ghada Ben Amor, Markus Pydde, Alexander Nagel, Saveria Germani, Lara Monica, Anja Schlör, Hannes Bauer, Jane Hornung, Michael Voetz, Yamen Dwai, Benjamin Scheer, Frauke Ringel, Omar Kamal-Eddin, Christoph Harms, Jonas Füner, Lorenz Adrian, Axel Pruß, Kai Schulze-Forster, Katja Hanack, Julian Kamhieh-Milz

**Affiliations:** 1 Institute of Transfusion Medicine, Charité-Universitätsmedizin Berlin, Corporate Member of Freie Universität Berlin, Humboldt-Universität zu Berlin, and Berlin Institute of Health, 10117 Berlin, Germany; 2 Preclinics Certified Products GmbH, 14482 Potsdam, Germany; 3 Wimedko GmbH, 12101 Berlin, Germany; 4 Sifin Diagnostics GmbH, 13088 Berlin, Germany; 5 Preclinics Gesellschaft für Präklinische Forschung mbH, 14482 Potsdam, Germany; 6 New/Era/Mabs GmbH, 14476 Potsdam, Germany; 7 CellTrend GmbH, 14943 Luckenwalde, Germany; 8 Department Environmental Biotechnology, Helmholtz Centre for Environmental Research—UFZ, 04318 Leipzig, Germany; 9 Center for Stroke Research Berlin with Department of Experimental Neurology, Charité-Universitätsmedizin Berlin, Corporate Member of Freie Universität Berlin, Humboldt-Universität zu Berlin, and Berlin Institute of Health, 10117 Berlin, Germany, 10117 Berlin, Germany; 10 Chair of Geobiotechnology, Technische Universität Berlin, 13355 Berlin, Germany; 11 Department of Biochemistry and Biology, University of Potsdam, 14476 Potsdam, Germany; 12 DHS—Diagnostic HealthCare Solutions GmbH, 13347 Berlin, Germany

**Keywords:** virus-like particle (VLP), SARS-CoV-2, vaccine, stable cell line, neutralizing antibodies

## Abstract

The rapid evolution of new SARS-CoV-2 variants poses a continuing threat to human health. Vaccination has become the primary therapeutic intervention. The goal of the current work was the construction of immunogenic virus-like particles (VLPs). Here, we describe a human cell line for cost-efficient and scalable production of immunogenic SARS-CoV-2 VLPs. The modular design of the VLP-production platform facilitates rapid adaptation to new variants. Methods: The N, M-, and E-protein genes were integrated into the genome of Expi293 cells (ExpiVLP_MEN). Subsequently, this cell line was further modified for the constitutive expression of the SARS-CoV-2 spike protein. The resulting cell line (ExpiVLP_SMEN) released SARS-CoV-2 VLP upon exposure to doxycycline. ExpiVLP_SMEN cells were readily adapted for VLP production in a 5 L bioreactor. Purified VLPs were quantified by Western blot, ELISA, and nanoparticle tracking analysis and visualized by electron microscopy. Immunogenicity was tested in mice. Results: The generated VLPs contained all four structural proteins, are within the size range of authentic SARS-CoV-2 virus particles, and reacted strongly and specifically with immunoserum from naturally infected individuals. The VLPs were stable in suspension at 4 °C for at least 10 weeks. Mice immunized with VLPs developed neutralizing antibodies against lentiviruses pseudotyped with the SARS-CoV-2 spike protein. The flexibility of the VLP-production platform was demonstrated by the rapid switch of the spike protein to a new variant of concern (BA.1/Omicron). The present study describes an efficient, scalable, and adaptable production method of immunogenic SARS-CoV-2 VLPs with therapeutic potential.

## 1. Introduction

Severe-Acute-Respiratory-Syndrome-Virus 2 (SARS-CoV-2) caused the disease COVID-19 and the recent pandemic [1,2]. In most cases, SARS-CoV-2 infection causes only mild symptoms, such as coughing, shortness of breath, or fever. However, severe symptoms, such as pneumonia, organ failure, and even death are associated with increasing age and pre-existing chronic conditions [3,4]. The rapid spread of SARS-CoV-2 is facilitated by efficient transmission by asymptomatic and pre-symptomatic individuals [5]. Globally, there have been more than 750 million confirmed cases of COVID-19, including almost 7 million deaths (WHO, 30 June 2023).

Structurally, the positive-sense, single-stranded RNA virus consists of the spike glycoprotein (S), nucleoprotein (N), membrane (M) and envelope (E) proteins [6,7]. The N-protein mediates the packaging of the genome into virions. The M- and E-proteins form the viral membrane, and their expression alone is sufficient for the spontaneous assembly and release of these viral membranous shells [8,9,10]. The protruding S-protein contains a high-affinity binding domain that interacts with human ACE2, which is the naturally occurring receptor for SARS-CoV-2 [11]. TMPRSS2 is a serine protease that cleaves the S-protein between the S1 and S2 subunit and significantly enhances the internalization of the virus from the plasma membrane [11,12,13].

Virus-like particles (VLPs) are macromolecular protein assemblies that mimic the structure of a virus. VLPs are non-infectious, as they do not contain replication-competent genetic material (RNA or DNA). Because VLPs are highly immunogenic, they have attracted considerable attention as vaccine candidates and as research tools to model mechanisms of infection [14,15,16]. VLPs can also be applied as antigens in diagnostic devices [17,18,19,20]. Since 2020, groups have produced SARS-CoV-2 VLPs in bacteria [21], yeast [22], plant [10,23], insect [24,25,26,27], and mammalian cells [8,18,28,29,30]. Most reported methods use transient transfection of plasmid DNA to deliver viral genes into target cells. Whereas transient transfection is relatively quick to adapt to new mutations, this method is expensive to scale up and prone to inducing differences between production charges. These drawbacks hamper the mass production of VLPs that would be necessary for the production of a vaccine candidate or antigen for diagnostic devices. Here, we describe a stably modified human cell line that produces immunogenic VLPs upon exposure to doxycycline, which minimizes economical and technical difficulties.

## 2. Materials and Methods

*Culturing of Expi293 cells:* The Expi293 Expression System Kit, composed of Expi293 suspension adapted cell line, Expi293 transfection reagents, and Expi293 culture medium was purchased from Thermo Fisher Scientific (# A14635, Dreieich, Germany). Cultivation and transfection of Expi293 cells were mostly performed according to the manufacturer’s instructions. Briefly, Expi293 cells were grown at 37 °C, 8% CO_2_ with 130–150 rpm on a Rotamax120 platform shaker (Heidolph Instruments, Schwabach, Germany) in Expi293 medium containing a final concentration of 100 units/mL penicillin and 100 µg/mL streptomycin. Cell diameter, the percentage of viable cells (viability), and the concentration of viable cells were measured using LUNA Cell Counter (Logos Biosystems, Anyang, South Korea). Cells were seeded at a density between 0.3 × 10^6^ and 0.5 × 10^6^ cells/mL and subcultured at a concentration of 3 × 10^6^ viable cells/mL after 3–5 days. For transfection, cells were precipitated for 5 min at 150× *g* and subsequently resuspended and adjusted in fresh media to a final concentration of approximately 3 × 10^6^ viable cells/mL and a viability exceeding 95%.

*Generation of SARS-CoV-2 VLPs from genetically modified cell lines:* As described previously [18], we used two genetically modified Expi293 suspension-adapted cell lines to produce virus-like particles. The human codon optimized M-(Gen Bank: QHD43419.1), E-(Gen Bank: QHD43418.1), and N-genes (Gen Bank: QHD43423.2) were stably integrated into the base cell line (Expi_MEN) using a PiggyBac transposon system for stable genetic integration [31]. Hygromycin and mCherry were used as selection markers. This cell line is readily adapted for the expression of the S-gene (Gen Bank: QHD43416.1) using EGFP and blasticidin as selection markers with a lentiviral vector. The S-gen includes the D614G mutation and the furin cleavage site was rendered unfunctional by the R683A and R685A substitution (FKO). The induction of VLP production is controlled by the tetracycline-responsive element promoter (TRE) [32] and can be activated by tetracycline or its analogs (e.g., doxycycline) [33,34]. The optimized concentration of 1 µg/mL doxycycline-hyclate (Sigma-Aldrich, Taufkirchen, Germany) was used to induce VLP production. On days 2, 3, and 4 after addition of doxycycline for VLP production, a volume of 20–30% of fresh medium, including doxycycline, was added to the cell culture. Approximately 96 to 120 h after induction at a viability of 40–60%, cell culture supernatants were processed as described below.

*Generation of VLPs by transient transfection:* Human codon optimized sequences of genes encoding the S and E structural proteins of SARS-CoV-2 were synthesized by BioCat GmbH (Heidelberg, Germany) and subcloned into the pcDNA3.1 Zeo(+) expression plasmid using the NheI 5′ and XhoI 3′ restriction site. The S-protein contained the D614G mutation and the furin-cleavage site was destroyed (FKO) by R683A and R685A substitution. A dual expression plasmid for the M- and N-proteins from the same ORF separated by a T2A self-cleaving peptide was generated by Vectorbuilder Inc. Cultivation and transfection of Expi293 cells was mostly performed according to the manufacturer’s instructions, except that 1 µg of total DNA per 10^6^ cells was used for transfection. pcDNA3.1-Spike(FKO), pcDNA3.1-E-Protein and pEXP-M+N-protein were transfected at a ratio of 6:2:3. Per 1 µg of DNA, 3.2 µL of Expi293 transfection reagent was used. Approximately, 96–120 h after transfection at a viability of 40–60%, cell culture supernatants were processed as described below.

*Processing of VLP containing cell culture supernatants:* Cell culture supernatants were harvested at a viability of 40–60%, generally 96–120 h after VLP production was induced by doxycycline or transient transfection. Subsequently, cell culture supernatants were cleared by centrifugation at 2000× *g* for 10 min followed by filtration with a 1.2 µm exclusion size Minisart NML filter (Sartorius Stedim Biotech GmbH, Göttingen, Germany) and thereafter by a 0.45 µm exclusion size Millex Low Binding Durapore (PVDF) syringefilter (Merk Millipore Ltd., Tullagreen, IE). Clarified cell culture supernatants were diafiltrated continuously with four times the initial volume of phosphate-buffered saline (PBS) pH 7.2 at a constant pressure of 0.124–0.165 kPa and 135 rpm using a Minimat EVO Tangential Flow Filtration System equipped with an Omega Membrane with a 300 kDa cut-off (Pall Corporation, Dreieich, Germany). 

VLPs were precipitated from the diafiltrated retentate or directly from the clarified supernatant by the addition of PEG-it Virus Precipitation Solution (System Biosciences, Palo Alto, CA, USA) at a volume ratio of 1:10 or 1:5. Supernatants were incubated at 4 °C on a rotating shaker for 24–48 h prior to precipitation at 1500× *g* for 30 min. The supernatant was carefully removed, and the VLP-containing pellet was resuspended with 0.05–0.1% of the initial volume with sterile PBS (pH 7.2). Resuspended pellets were kept at 4 °C for short-term (1–3 weeks) or at −80 °C for long-term storage. The precipitation of VLPs with PEG was developed further because it was more practical to scale and showed better yields of spike and nucleoprotein in initial experiments than ultracentrifugation and filtration-based concentration of VLPs from cell culture supernatant.

*Fluorescence microscopy:* Suspended cells were loaded onto LUNA Cell Counting Slides (Logos Biosystems) and imaged using an Olympus IX81 microscope equipped with a LedHUB illumination system (Omicron-Laserage Laserprodukte GmbH, Rodgau, Germany). Fiji ImageJ software version 1.53f51 [35] was used to quantify the proportion of transduced cells expressing EGFP or mCherry.

*Total protein concentration:* Total protein concentrations were analyzed with the BCA Protein Assay Kit according to the manufacture’s instruction (Thermo Fisher Scientific, Wilmington, DE, USA) using bovine serum albumin (BSA) as a standard. Predefined dilutions were applied in duplicate or triplicate. The absorbance was quantified at 562 nm on an Infinite PRO NanoQuant (Tecan Group Ltd., Männedorf, Switzerland) microplate reader using clear 96-well plates.

*Nanoparticle tracking analysis (NTA):* NTA was used to determine the size and concentration of VLPs. NTA measurements were performed using a NanoSight LM10 instrument (NanoSight, Amesbury, UK) consisting of a conventional optical microscope, high-sensitivity sCMOS camera, and an LM10 unit equipped with a 488 nm laser module. Samples were injected into the LM unit via a nanosight syringe pump with a constant flow rate of 50 µL/min using a 1 mL sterile syringe. Sample dilutions of 1:2000 to 1:5000 were observed to result in an effective particle concentration suitable for NTA analysis (10^8^ to 2.5 × 10^9^ particles/mL). The capturing settings (shutter and gain) and analyzing settings were adjusted manually and kept constant between all samples that were recorded on the same day. NTA software (NTA 3.2 Dev Build 3.2.16) was used to capture three 30 s videos per sample.

*Electron microscopy:* Four days after doxycycline induction, 5 × 10^6^ VLP-producing cells were pelleted at 300× *g* for 5 min. The cell pellet was resuspended in a fixation solution containing 2.5% glutaraldehyde (Serva, Heidelberg, Germany) in 0.1 M sodium cacodylate buffer (Serva, Heidelberg, Germany) for 30 min at room temperature, followed by storage at 4 °C. Samples were postfixed with 1% osmium tetroxide (Science Services, München, Germany) and 0.8% potassium ferrocyanide II (Merck, Darmstadt, Germany) in 0.1 M cacodylate buffer for 1.5 h and embedded in 1% agarose (Sigma-Aldrich). After cutting the agarose in smaller blocks, the samples were dehydrated in a graded ethanol series and embedded in Epon resin (Serva, Heidelberg Germany). Finally, ultrathin sections of the samples (70 nm) were stained with 4% uranyl acetate and Reynolds lead citrate (Reynolds 1963) (Merck). The examination was carried out with a Zeiss EM 906 electron microscope at 80 kV acceleration voltage (Carl Zeiss, Oberkochen, Germany) equipped with a slow scan 2K CCD camera (TRS, Moorenweis, Germany). Images were processed using Fiji ImageJ version 1.53f51 [35]. Some particles had a slight oval shape. Therefore, we defined the diameter as the mean between the horizontal and transverse axis of each particle.

*Mass spectrometric protein detection:* Coomassie-stained protein bands were cut from the gel, destained, reduced with dithionite, cysteine-derivatized with iodoacetamide, and trypsin-digested overnight within the gel piece. Subsequently, peptides were extracted from the gel piece, and desalted with ZipTips, all as described previously [36]. Peptides were analyzed by nano-liquid chromatography on an Acclaim PepMap100 C18 column (Thermo Scientific, Dreieich, Germany) on an Orbitrap Fusion mass spectrometer (Thermo, Dreieich, Germany) at a mass resolution of 120,000 for precursor ions. Fragmentation was accomplished with higher-energy collisional dissociation and fragments were analyzed in the Orbitrap analysator at a resolution of 60,000 [37]. Data analysis was accomplished with ProteomeDiscoverer version 2.4 using a database of VLP-sequences plus their decoy sequences for calculation of false discovery rates as search space. Estimation of quantitative values was performed from precursor intensities, with the Minora node implemented in ProteomeDiscoverer 2.4.

*ELISA (Enzyme-linked Immunosorbent Assay):* S-protein (Sino Biological Inc, Beijing, China, #40689-V08B), N-protein (charge 2020/20.7/2, 0.4 mg/mL stock, new/era/mabs GmbH, Potsdam, Germany) or SARS-CoV-2 VLPs were immobilized as an antigen on a Nunc Polysorb 96-well microtiter plate in 100 µL carbonate buffer per well overnight at 4 °C. Free binding sites were blocked with 250 µL per well of PBS containing 1% BSA for 45 min at room temperature. After washing with PBS, rabbit monoclonal antibodies against the spike protein (1:2000, 40689-V08B, Sino Biological Inc., Beijing, China) and nucleoprotein (1:5000, 40143-R019, Sino Biological Inc, Beijing, China), or human serum (1:50), were incubated with the immobilized antigen in 100 µL PBS containing 0.05% Tween-20 (PBS-T) and 1% BSA at room temperature for 1 h. After an additional washing step, plates were incubated at room temperature with a horseradish peroxidase (HRP)-conjugated goat anti-rabbit (#111-035-045), goat anti-mouse (#115-035-146), or goat anti-human (#109-035-088) IgG antibody (Dianova GmbH, Hamburg, Germany) diluted 1:10,000 in PBS-T containing 1% BSA. These secondary antibodies were raised against whole molecule IgG but also react with the light chains of other immunoglobulin molecules of the indicated species. The colorimetric reaction using 100 µL tetramethylbenzidine (TMB, Carl Roth GmbH, Karlsruhe, Germany) as substrate was stopped with 100 µL of 0.125 M H_2_SO_4_ per well after 10–30 min. Absorbance was measured using an Epoch microplate reader at ʎ = 450 nm and subtracted by the absorbance of the reference wavelength at ʎ = 620 nm. For the quantitative approximation of N- and S-protein concentrations in VLP samples, the linear range of a 2-log dilution series (triplicate) of the respective proteins was taken as a reference.

*Western blot:* Samples were prepared according to the NuPAGE Technical Guide of Invitrogen. Briefly, after denaturation in NuPAGE LDS sample buffer with DTT 50 mM for 10 min at 70 °C, samples and markers were run on a Novex bis-tris gradient gel (4–12%, Thermo Fischer Scientific, Dreieich, Germany) using NuPAGE MOPS SDS running buffer and blotted subsequently on a Novex 0.45 µm nitrocellulose membrane (LC2001, Thermo Fischer Scientific, Dreieich, Germany). Membranes were blocked in PBS containing 1% BSA and subsequently incubated with a rabbit-anti-nucleoprotein primary antibody (1:5000, 40143-R019, Sino Biological Inc, Beijing, China) or human serum in PBS-T containing 1% BSA at 4 °C overnight on a shaker. Human serum from a double-vaccinated individual (AstraZeneca) or convalescent serum (PCR-confirmed SARS-CoV-2 infection) was used to detect the S-protein alone or S- and N-proteins, respectively. High levels of anti-S or anti-S and anti-N-protein IgGs of the respective human sera were confirmed by ELISA. HRP-coupled goat anti-human (#109-035-088) or goat anti-rabbit (#111-035-045) IgG antibodies (Dianova GmbH, Hamburg, Germany) were incubated 1:10,000 in PBS-T for 3 h at room temperature. These secondary antibodies were raised against whole molecule IgG but also react with the light chains of other immunoglobulin molecules of the indicated species. Proteins were detected by chemiluminescence using respective kits from Biozym Scientific GmbH (Hessisch Oldendorf, Germany) and submitted to image analysis via Fiji ImageJ version 1.53f51 [35].

*Analytical gel filtration:* Analytical size exclusion chromatography was performed on an Äkta Pure 25 using Superdex 200 Increase 5/150 GL-Column. A flow rate of 0.3 mL/min was used throughout the experiment. The column was equilibrated with PBS containing 0.04% sodium azide as running buffer. All SARS-CoV-2-VLP-containing samples were vortexed gently. Cell culture supernatants were then filtered through 0.2 µm cut-off Millex Low Binding Durapore (PVDF) syringe filter (Merk Millipore Ltd., Tullagreen, Ireland). Resuspended SARS-CoV-2-VLP-containing pellets were centrifuged briefly at 16,000× *g* for 10 min immediately before loading onto the column. Calibration of the retention time and protein size was performed using a standard containing thyroglobulin (MW: 669.0 kDa), aldolase (MW: 158.0 kDa), ovalbumin (MW: 43.0 kDa), carbonic anhydrase (MW: 29.0 kDa), and ribonuclease A (MW: 13.7 kDa). A specific peak at 1.18 mL was observed only in VLP-containing samples. The size was calibrated using the polynomial calibration function (Y = −361.27 x^3^ + 2686.5 x^2^ – 6612.7 x + 5409).

*Immunization of mice:* All animal experiments were conducted according to the German federal animal welfare legislation. Virus-like particles were tested in two concentrations (12.5 µg and 50 µg) in four cohorts of each eight female mice. An additional cohort of four mice was treated with NaCl 0.9% as control. On day 0, all animals received an intramuscular injection of the respective test substances into the right gastrocnemius muscle. On day 21, two cohorts of animals received a boost intramuscular injection into the left gastrocnemius muscle. The other three groups received buffer. On day 43, the animals were sacrificed by exsanguination (for serum preparation). 

*Neutralization assay:* Antisera from animals treated with high dose (50 µg) of VLPs were diluted 1:20 as a starting dilution; seven further dilutions of the samples were made in 1:3 steps (up to 1:21,870 dilution) in a 96-well plate (Corning Life Science Plastic, Bedford, MA, USA). SARS-CoV-2 Wuhan variant pseudotyped lentiviral particles were produced in house or purchased (220810SLVP48, VectorBuilder, Guangzhou, China) and diluted 1:5000. All dilutions were made with cell culture medium DMEM (Biowest, Nuaillé, FR) containing 10% fetal bovine serum (FBS), 1% penicillin-streptomycin (Pen-Strep) and 10 µM HEPES. The neutralization step was performed mixing SARS-CoV-2 pseudotyped lentiviral vectors with the samples (ratio 1:1) and incubated for 1 h, 37 °C. After the incubation step, a cell suspension of 293T cells ectopically expressing hACE2 and hTMPRSS2 (293T hACE2-hTMPRSS2 cells, VectorBuilder, Guangzhou, China) were added to the neutralization mix (ratio 1:1), transferred with Integra Assist Plus (Integra, Berlin, Germany) in a 384-well white plate, clear bottom (Corning Life Science Plastic, Bedford MA, USA), and incubated for 72 h, 37 °C. After the incubation, the medium was removed from the wells, and cells were lysed with Lysis Juice (PKJ Biotech, Kleinblittersdorf, Germany) and kept on a plate shaker for 30 min at 200 rpm, RT. Finally, the injection of D-Luciferine substrate for firefly luciferase (Beetle Juice Fluid, PKJ Biotech, Kleinblittersdorf, DE) and the reading of the plates at 560 nm were executed with the plate reader Mithras LB 940 (Berthold technologies, Bad Wildbad, Germany).

*Data evaluation and statistical analysis:* Statistical analysis was performed using Prism 8 (GraphPad, San Diego, USA). *p* values of <0.05 were considered statistically significant. Differences between the groups were tested with ANOVA or respective nonparametric methods (Kruskal–Wallis-test), followed by multiple comparisons (Dunnet’s or Dunn’s tests). Significance levels are marked by asterisks, where * corresponds to *p* < 0.05, **—*p* < 0.01 and ***—*p* < 0.001.

## 3. Results

*Characterization of an inducible SARS-CoV-2 VLP producer cell line*

**Figure 1:**
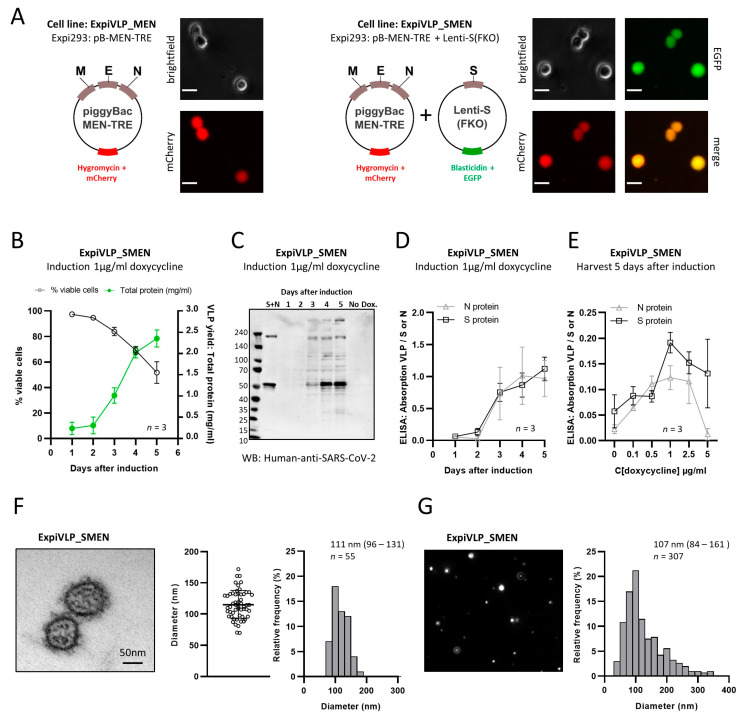
Development of a cell line for the inducible production of SARS-CoV-2 VLPs containing all four major structural proteins. (**A**) Illustration of genetic constructs and fluorescent images of Expi293 cells. The presence of both fluorescent markers (EGFP and mCherry) indicates the presence of all four structural proteins after antibiotic selection. (**B**) Inverse relationship between cellular health and protein precipitated from daily samples of the cell culture supernatant of three individual batches after induction of VLP production with 1 µg/mL doxycycline. (**C**) Western blot of protein precipitated from daily samples after induction of VLP production with 1 µg/mL doxycycline. Human serum from a previously infected individual detected SARS-CoV-2 spike- and nucleoproteins from the third day after doxycycline stimulation (**D**,**E**) ELISA using monoclonal rabbit antibodies against the spike- or nucleoprotein. The harvested VLPs were coated at a concentration of 5 µg/mL to the solid phase of a microtiter plate. (**D**) From daily samples after doxycycline stimulation. (**E**) VLP production was induced with different concentrations of doxycycline and samples harvested on the 5th day after induction. (**F**) Quantification of VLP-diameter by electron microscopy. (**G**) Quantification of VLP-diameter by nanoparticle tracking analysis (NTA).

Our primary goal was to generate a scalable and adaptable cell line for the inducible production of SARS-CoV-2 VLPs. Here, we devised a two-step strategy to genetically modify Expi293 cells for the expression of four SARS-CoV-2 structural proteins. In the first step, the N-, M-, and E-protein genes were stably integrated into Expi293 cells. Hygromycin and mCherry genes were used for the selection of positive clones (Figure 1A). Cell counts obtained with a fluorescence microscope revealed that 99.56 ± 0.63% of the cells expressed the marker protein mCherry after four passages of culturing the transfected cells in the presence of 20 µg/mL hygromycin. This cell line (ExpiVLP_MEN) was used to generate control SARS-CoV-2 VLPs that contain the N-, M-, and E-proteins but do not contain the spike protein (“smooth VLPs”). The ExpiVLP_MEN cell line served as the precursor cell line for the transduction with a lentiviral vector to achieve the expression of a modified SARS-CoV-2 spike protein (Figure 1A) in addition to the N-, M-, and E-protein. The spike protein contained the D614G mutation. The furin cleavage site between the S1 and the S2 domain was knocked out (FKO). After four passages of culturing the transduced cells with 10 µg/mL blasticidin and 20 µg/mL hygromycin, 98.36 ± 2.32% of the cells expressed the fluorescent marker protein EGFP in addition to mCherry. The typical viability of 95% to 99% indicated that the cells adapted well to the presence of both antibiotics. qPCR [38,39] was used to calculate that on average 2.39 vector copies of the spike protein coding lentiviral construct per cell had been integrated into the host cell genome.

Next, we established optimal conditions to induce VLP production from the ExpiVLP_SMEN producer cell line. VLP production was induced in three individual batches with doxycycline at the optimized concentration of 1 µg/mL (Figure 1B–E). Cell concentration and viability was monitored for five consecutive days by an automated quantification of trypan-blue-stained cells and a 10 mL sample of the cell culture supernatant was taken daily for analysis. After three days of culturing ExpiVLP_SMEN in the presence of 1 µg/mL doxycycline, the trypan blue staining indicated that the cells stopped proliferating (Figure 1B). The viability dropped continuously from 97.43 ± 0.78% on day 1 to 51.7 ± 8.47% on day 5 after induction. VLPs from the cell culture supernatant were precipitated with PEG-it Virus Precipitation Solution according to the manufacturer’s protocol. The change in cellular fitness was accompanied by a steep increase in the yield of VLPs, which was quantified as total protein by bicinchoninic acid assay (BCA assay). The spike protein and the nucleoprotein were identified in VLP-containing pellets from the third to the fifth day after doxycycline induction by Western blot, using human convalescent serum to detect SARS-CoV-2 proteins (Figure 1C). The same result was achieved by ELISA using monoclonal antibodies to detect the nucleoprotein and the spike protein (Figure 1D). SARS-CoV-2 proteins were not detected in precipitates from cell culture supernatant of ExpiVLP_SMEN cells that were cultured in parallel in the absence of doxycycline. To verify the expression of the M- and E-protein, we searched for SARS-CoV-2-specific peptides in the mass spectra from harvested and trypsin-digested VLP proteins. The presence of all four SARS-CoV-2 structural proteins was demonstrated by the identification of several unique peptides that covered large parts of the sequence of the S- (58%), N- (75%), M- (26%), and E-protein (39%) (Table 1). To confirm the release of particles with a virus-like appearance, samples from the fourth day after doxycycline induction were fixed with glutaraldehyde, embedded in Epon resin, stained with 4% uranyl acetate, and Reynolds lead citrate, and imaged using a transmission electron microscope (Zeiss EM 906). The median diameter of the SARS-CoV-2 VLPs from the ExpiVLP_SMEN cell line was 111 nm 95% CI [96–131, *n* = 55 particles] (Figure 1F). By applying cell culture supernatant from the same sample to nanoparticle tracking analysis (NTA), we found a particle diameter of 107 nm 95% CI [84–161, *n* = 307 particles] (Figure 1G). We concluded that our SARS-CoV-2 VLPs are of comparable diameter to authentic wild-type SARS-CoV-2 (60–140 nm) [2,40,41].

*Comparison of VLPs produced by transient transfection and induction of ExpiVLP_SMEN*

**Figure 2:**
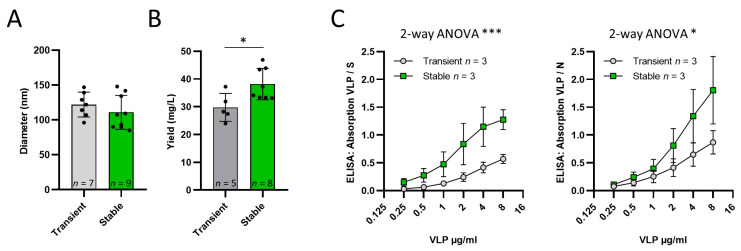
Comparison of VLPs produced by transient transfection and by the ExpiVLP_SMEN cell line. (**A**) Cell Diameter assessed by NTA. (**B**) VLP yield per ml. (**C**) Anti-spike protein and anti-nucleoprotein ELISA comparing the relative absorption generated by VLPs in a 2-Log dilution series of the coating concentration normalized to the absorption of 0.5 µg/mL spike- or nucleoprotein. *—*p* < 0.05 and ***—*p* < 0.001.

SARS-CoV-2 VLPs were produced by transient transfection of Expi293 cells with an optimized protocol according to our previous publication [17]. Data from eight independent VLP productions from the ExpiVLP_SMEN cell line (M = 38.16, SD = 5.68), compared to five independent productions by transient transfection (M = 29.73, SD = 5.038), demonstrated a significantly higher VLP yield milligram per liter of cell culture volume, t(11) = 2.712, *p* = 0.0202 (Figure 2B). Nanoparticle tracking analysis showed no measurable difference in particle size (t(14) = 1.003, *p* = 0.3327) of VLPs generated by transient transfection (M = 122.0, SD = 17.93, *n* = 7) and by the ExpiVLP_SMEN cell line (M = 110.9, SD = 24.41, *n* = 9). The VLPs from both production methods were coated as a two-fold dilution series to the solid phase of a microtiter plate. ELISAs were performed to analyze the effect of VLP production method on the normalized absorption. In addition, 0.25 µg/mL of the spike protein or nucleoprotein was used as a reference. Two-way ANOVA revealed that VLPs from the ExpiVLP_SMEN cell line incorporated a significantly larger proportion of spike protein (F(1, 24) = 19.73, *p* = 0.0002, *n* = 3) and nucleoprotein (F(1, 24) = 6.133, *p* = 0.020, *n* = 3) than VLPs generated by transient transfection (Figure 2C).

*Purification of VLPs from the ExpiVLP_SMEN cell line by tangential-flow filtration and PEG precipitation*

**Figure 3:**
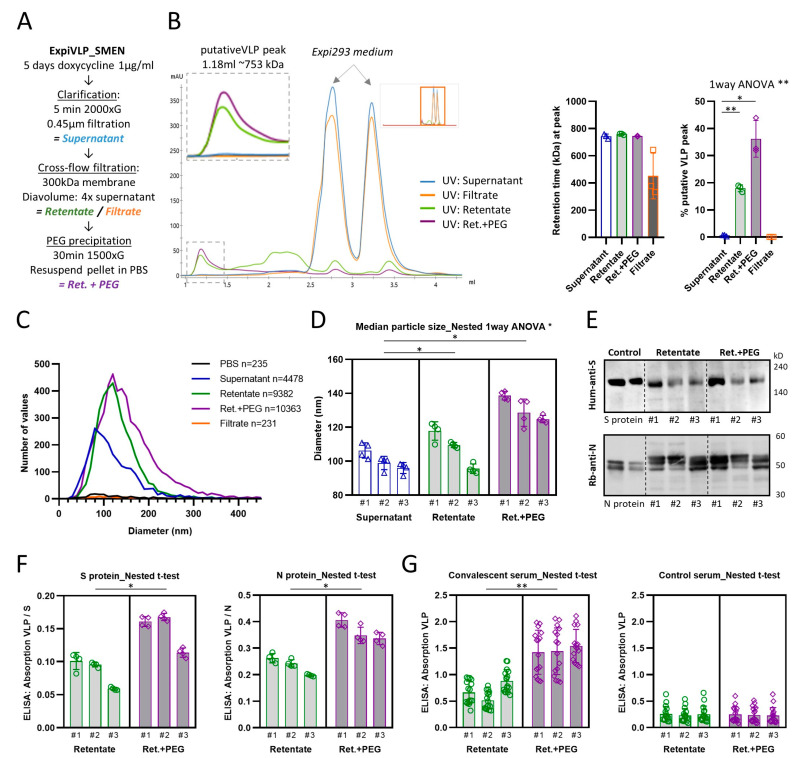
Cross-flow filtration followed by PEG precipitation improves SARS-CoV-2 VLP purity and specific protein content. (**A**) Workflow of VLP purification. (**B**) Example trace and UV quantification of analytical gel filtration of various samples from three individual batches during the purification workflow. VLP and unspecific peaks are highlighted. (**C**) Size distribution (additional graph in Figure S1) and (**D**) quantification of various samples during the purification workflow. (**E**) Western blot showing the presence of spike- and nucleoprotein in VLPs from three individual batches (complete membrane in Figure S2). (**F**) Spike- and nucleoprotein ELISA of the retentate and the precipitation from the retentate of three individual batches. (**G**) ELISA measuring the reactivity of the retentate and the precipitation from the retentate of three individual batches with convalescent and control serum. *— *p* < 0.05 and **—*p* < 0.01.

To improve the purification of VLPs harvested from the supernatant of ExpiVLP_SMEN cells exposed to doxycycline (1 µg/mL) for five days, we introduced a cross-flow filtration step using a Minimate tangential-flow filtration system. The cell culture supernatant was cleared by centrifugation and sterile-filtered prior to diafiltrating with PBS. Four times the initial volume was fed continuously to the supernatant. A 300 kDa Minimate tangential flow filtration capsule with an approximate pore size of 35 nm was used to separate the VLPs from smaller molecules in the supernatant. Filtration was performed at 0.124–0.165 kPa which generated a mean flow rate of 4.79 ± 1.01 mL/min (SD) across the three batches that were systematically analyzed. Subsequently, VLPs were precipitated with PEG-it Virus Precipitation Solution (System Biosciences) and pellets resuspended in PBS (Figure 3A). Intermediate samples were taken and stored at 4 °C with sodium azide until they were analyzed by analytical gel filtration, nanoparticle tracking analysis, Western blot, and ELISA. The analytical gel filtration of the cell culture supernatant from all three analyzed preparations suggested that the VLP elution peak was at a retention time of 1.186 ± 0.008 mL (SD). The same peak was found in the retentate after diafiltration (M = 1.177, SD = 0.002 mL) and the PEG-it precipitates that were prepared from the retentate (M = 1.185, SD = 0.001 mL) (Figure 3B). These retention peaks related to a calibrated mean molecular weight of 743.2 ± 13.8 kDa for the VLP peak of the supernatant, 759.1 ± 4.2 kDa for the retentate, and 744.2 ± 2.5 kDa for the PEG-it precipitate from the retentate. There was no significant difference between the calibrated molecular weights (F(1.116, 2.231) = 3.692, *p* = 0.1841). Both purification steps significantly increased the proportion of the VLP peak which correlated to the purity of the sample (one-way ANOVA: F(1.002, 2.003) = 49.80, *p* = 0.0194) (Figure 3B). The purification steps increased the mean percentage of the VLP peak from 0.55 ± 0.22% in the supernatant to 17.98 ± 1.05% in the retentate after diafiltration. PEG-it precipitation of the VLP from the retentate doubled the VLP proportion (M = 36.16, SD = 5.51%). We found that the VLP peak was significantly greater (paired t-test: *p* = 0.0261) in PEG-it precipitated VLP from the retentate as compared to a direct precipitation from the cleared cell culture supernatant (M = 7.72, SD = 2.02%). However, we still noticed that small but consistent peaks, which represent the Expi293 media alone, were present in the diafiltrated retentate and PEG-it precipitated VLP from the retentate. We concluded that larger diafiltration volumes would be beneficial.

Next, we analyzed whether the purification steps had an impact on the size of the particles which was determined by nanoparticle tracking analysis (Figure 3C,D). Indeed, nested one-way ANOVA revealed a significant difference (F(2, 6) = 0.37, *p* = 0.0113, *n* = 3 batches) between the median particle diameter of the cleared supernatant (M = 99.61, SD = 3.24), the retentate (M = 108.1, SD = 1.38), and the PEG-it precipitation from the retentate (M = 132.2, SD = 2.09). Interestingly, we found that VLPs that were precipitated with PEG-it from the retentate incorporated significantly larger amounts of spike protein (nested t-test: *p* = 0.0441) and nucleoprotein (nested *t*-test: *p* = 0.0108) (Figure 3F). This was also reflected in a significantly higher reactivity of the PEG-it precipitated samples with convalescent serum from 16 individuals that recovered from a previous SARS-CoV-2 infection (nested t-test: *p* = 0.0023) (Figure 3G). No difference was observed in the reactivity with control serum (nested *t*-test: *p* = 0.7846). The demonstrated improvements in purity and reactivity of VLPs precipitated from diafiltrated supernatant established this method as our preferred preparation protocol.

*Quantification of N- and S-protein contend of purified SARS-CoV-2 VLPs*

The combination of nanoparticle tracking and a quantitative ELISA was used to further our understanding of the molecular composition of the VLPs that were purified by cross flow filtration and precipitated with PEG-it. Samples from three independent batches were subjected to an in-house ELISA to quantify the concentration of N- and S-protein against a two-fold serial dilution of the reference protein (Table 2). The VLP samples contained a mean N-protein concentration of 0.16 ± 0.02 µg/µL (SD) and a mean S-protein concentration of 0.15 ± 0.02 µg/µL (SD). Taking the number of VLPs per microliter (M = 1.85 × 10^9^, SD = 3.14 × 10^8^) and the predicted molecular weight of the N-protein (45.63 kDa) and S-protein (140.95 kDa) into account, we calculated the mean number of 1174 ± 275.50 N-proteins and 121.51 ± 36.15 trimeric S-proteins per VLP.

*Large-scale production of VLPs in a 5 L bioreactor*

The ExpiVLP_SMEN cell line was transferred to a roller bottle cell culture system and cultivated up to a density of 1.35 × 10^6^ cells/mL and a viability of 91%. Subsequently, this starting culture was transferred to a 5 L bioreactor and cultivated with continuous supply of fresh serum containing media up to the maximal volume of the bioreactor, which was reached after four days. Cells had a density of 1.5 × 10^6^ cells/mL and were fed continuously for another four days to further increase cell density. The induction of VLP production by the addition of doxycycline was initiated at a cell density of approximately 1 × 10^7^ cell-agglomerations per milliliter. Of note, the exact concentration of cells could only be approximated because cells agglomerated into spherical units of five to ten cells. For another three days, cells were fed continuously with doxycycline containing fresh medium and harvested on day seven after induction. Using nanoparticle tracking analysis, we determined, that the total harvest of 4.7 L cell culture supernatant contained 7.68 × 10^11^ ± 3.16 × 10^10^ (SD) particles per milliliter. The purified VLP-containing samples contained 1.85 ± 0.01 (SD)% nucleoprotein and 1.99 ± 0.06 (SD)% spike protein, as determined by ELISA. The protein concentration was 1.35 ± 0.04 (SD) milligram per mL.

*SARS-CoV-2 VLPs are immunogenic in mice and elicit a neutralizing antibody response*

**Figure 4:**
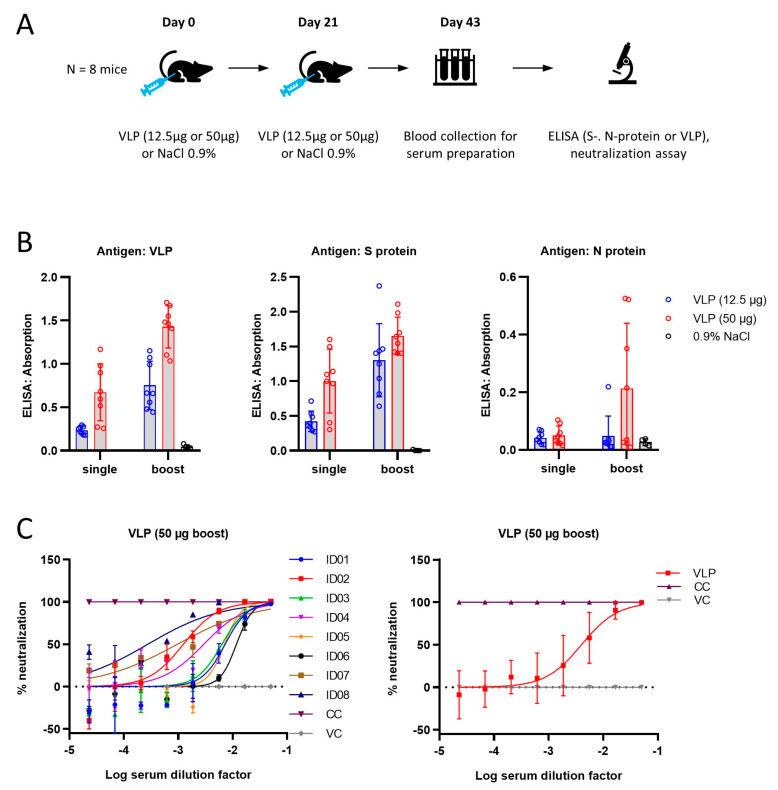
SARS-CoV-2 VLPs are immunogenic in mice. (**A**) Cartoon depicting the immunization protocol. (**B**) ELISA results. Antigens were coated to the microtiter plate at a concentration of 5 µg/mL VLP, 0.5 µg/mL spike protein, and 1 µg/mL nucleoprotein. Secondary antibody anti-mouse IgG (H+L). (**C**) Neutralization assay using pseudotyped lentiviral particles expressing firefly luciferase.

In order to establish whether the VLPs produced by the ExpiVLP_SMEN cell line are suitable as a vaccine candidate, we conducted an initial study in mice (Figure 4A). Control solution (NaCl 0.9%) or VLPs were injected at a low dose (12.5 µg) or a high dose (50 µg) into the gastrocnemius muscle of five groups of mice. Two groups received a single prime injection of the high or the low dose on day zero. The control group, a low-dose group, and a high-dose group received a prime dose on day zero and a second booster dose on day 21. Blood was collected on day 43 and analyzed for serum antibody levels by ELISA (Figure 4B). Two-way ANOVA comparing the optical density values between the high or low dose with the number of injections revealed a positive effect of the number of doses in the VLP ELISA (F(1, 28) = 40.09, *p* < 0.0001), the spike protein ELISA (F(1, 28) = 11.75, *p* = 0.0019), and the nucleoprotein ELISA (F(1, 28) = 4.215, *p* = 0.0495). The concentration of VLPs had a positive effect in the VLP-ELISA (F(1, 28) = 52.59, *p* < 0.0001) and the spike protein ELISA (F(1, 28) 31.97, *p* < 0.0001). There was no significant interaction between the number of doses and the injected dose (*p* > 0.05). Subsequently, the serum samples of the eight animals that received two injections with 50 µg of VLPs were subjected to a neutralization assay (Figure 4C). The calculation of the relative neutralization activity (percentage of luciferase signal) of the tested sera was made by comparing the signal of cells transduced with lentiviruses pseudotyped with the SARS-CoV-2 spike protein (B.1, Wuhan) in the presence of the serum to the luciferase signal detected in a cell-only control (CC, 293T-hACE2-hTMPRSS2 cells incubated with medium only, 100% neutralization) and a virus-only control (VC, cells incubated with pseudovirus without any serum, 0% neutralization). The mean serum dilution that corresponded to a 50% neutralization was 793.15 ± 397.28 (SEM). The results from the neutralization assay are in line with the immune response detected by ELISA.

*SARS-CoV-2 VLPs are stable at 4 °C for 10 weeks in solution*

The distribution of a vaccine at the appropriate temperature is critical and caused delays at the beginning of the recent pandemic. Therefore, we next investigated the stability of VLPs produced from the ExpiVLP_SMEN cell line at different temperatures. In an initial experiment, VLPs were resuspended in sterile PBS to a concentration of 2.08 mg/mL, analyzed by nanoparticle tracking analysis and ELISA and then stored in cryogenic tubes at −80 °C, −20 °C, +4 °C, +20 °C or +36 °C (Figure S3). After four-week storage, we found a prominent reduction in the particle concentration in samples that were stored at −20 °C (−47.2%), +20 °C (−35.8%) or +36 °C (−76.8%) and a significant loss of reactivity with human convalescent serum when compared to the initial data from before the stability test (ANOVA with Dunnett’s multiple comparison vs. Week 1, −20 °C *p* = 0.0009, +20 °C *p* = 0.0190, +36 °C *p* = 0.0001). In contrast, samples stored at −80 °C or +4 °C showed only minor changes in the nanoparticle tracking analysis and were consistently detected by human convalescent serum (ANOVA with Dunnett’s multiple comparison vs. Week 1, −80 °C *p* = 0.9997, +4 °C *p* = 0.4960).

**Figure 5:**
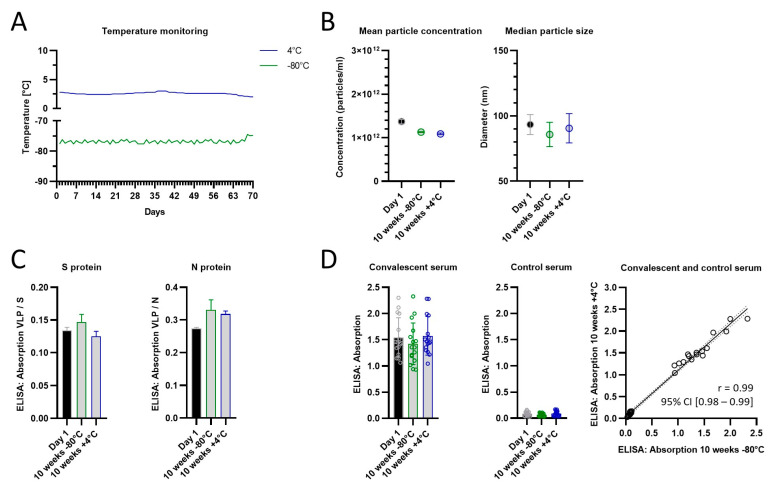
SARS-CoV-2 VLPs are stable at 4° and −80 °C in solution for at least 10 weeks. (**A**) Storage temperature monitored by a data logger. The quality of VLPs was assessed after 10 weeks by (**B)** NTA, (**C**) spike- and nucleoprotein ELISA and (**D**) by the reactivity of the VLPs with human convalescent (*n* = 16) and control serum (*n* = 16).

In a follow-up stability test, we compared the storage in the fridge and a deep freezer and extended the storage time with a new batch of VLPs to 10 weeks (Figure 5). Encouragingly, we detected no differences in particle size or concentration, protein composition (spike- and nucleoprotein), or reactivity with convalescent serum after 10 weeks when compared to week 1. There was a strong correlation in the reactivity between +4 °C or −80 °C storage for 10 weeks, which suggests that for this time period both methods preserve the VLPs equally well.

*Rapid adaptation of ExpiVLP_MEN with the BA.1 spike protein*

The VLP-producing cell line developed here was constructed as a modular system so that the base-cell line contains the M-, E-, and N-protein and lacks the S-protein. Therefore, we could rapidly add a new spike protein (Omicron BA.1) by infecting the parental cell line (ExpiVLP_MEN) with a newly constructed lentivirus for the dual expression of the BA.1 spike protein and puromycin selection marker. qPCR [38,39] was used to calculate that on average 1.8 vector copies of the BA.1 spike protein coding lentivirus per cell have been integrated into the host cell genome. Subsequently, we used the established protocol to induce VLP production. That way, we were able to generate the adjusted SARS-CoV-2 BA.1-Spike VLPs within a few weeks. Using the established anti N-protein ELISA and an in-house ELISA with the BA.1 spike protein as a reference, we determined that the SARS-CoV-2 BA.1-Spike VLPs contained 5.51 ± 0.12% N-protein and 3.26 ± 0.10% BA.1 spike protein.

## 4. Discussion

In the current study, we describe a platform technology for the reproducible and adaptable production of VLP-based vaccine candidates for COVID19. We have generated a bioreactor-compatible mammalian cell line that produces SARS-CoV-2 VLPs upon exposure to doxycycline. The VLPs closely mimic the authentic SARS-CoV-2 and are immunogenic in mice. Furthermore, the design of our genetic construct allows the rapid adaption of the cell line without affecting down-stream processes.

The SARS-CoV-2 VLPs produced by our system are structurally intact, are within the size range of the authentic virus, and contain all four major structural proteins without tags or deletions. The only modification is the destruction of the furin-cleavage site between the S1 and S2 subdomain to avoid loss of S1 during the purification steps. This is one of the few studies that directly validates the presence of the M- and E-protein in their VLPs and, to our knowledge, the only study that quantified the number of nucleoproteins and spike proteins per particle. We found approximately 1100 nucleoproteins and 120 spike trimers per VLP which are within the reported ranges for natural viruses of SARS-CoV-2 and the closely related SARS-CoV. From the structural data on ribonucleoprotein complexes reported by two groups, it was approximated that SARS-CoV-2 contains at least 300 to 600 nucleoproteins per virion [7,40]. The estimates for SARS-CoV range from 730 to 2200 copies per virion [42]. Structural data for S-protein trimers per SARS-CoV-2 virion range from 25 to 127 copies per virion for SARS-CoV-2 [40,41] and approximately 100 trimers for SARS-CoV [42].

The generated inducible cell line is the simplest production method for SARS-CoV-2 VLPs that has been described today. ExpiVLP_SMEN cells can be grown to the required volume and density. Subsequently, VLP production is induced in one simple step by the addition of doxycycline. The fact that SARS-CoV-2 proteins were not detected in the absence of doxycycline indicates low leak expression of the inducible promoter and the absence of the M- and E-proteins which are necessary for VLP assembly and release [8,10,28]. Here, we report a yield of 7.68 × 10^14^ particles per liter in the unpurified supernatant from the fed production, or 3.2 × 10^13^ purified particles and 33.7 mg protein per liter of cell culture from the shaker culture. The purified samples contain 160 mg/mL nucleoprotein and 150 mg/mL spike protein and 1.85 × 10^12^ particles per milliliter. With these specifications, our production system exceeds the yield of all, to our knowledge, reported production systems in mammalian or insect cells.

Mammalian cell lines have the advantage of the correct glycosylation, which is important for the spike protein, instead of the simpler glycosylation in insect cells or no glycosylation in bacterial cells. Roy et al. 2021 used a stable mammalian platform to generate chimeric VLPs from the Moloney murine leukemia virus (MLV) that were pseudotyped with the SARS-CoV-2 spike protein [29]. They estimated a titer of purified particles of 10^5^ infectious units per milliliter and that approximately 1.25 μg/mL S2 equivalent was integrated into their VLPs. Yilmaz et al. 2021 used transient transfection of HEK293 cells to generate VLPs that contained all four major structural proteins of SARS-CoV-2 [30]. The total protein yield was estimated at 25 mg/L of cell culture. For the production of VLPs in insect cells, the reported yields range from approximately 5 × 10^11^ purified particles per liter of cell culture using a baculoviral system [26] up to approximately 10^13^ particles per liter of unpurified cell culture supernatant using a baculovirus-free transient expression system [25].

We could not compare the VLP yield to the reported production in yeast [22] or plants [10] because the data were not disclosed. Mohsen and colleagues 2022 reported a yield of 250 mg per liter fermentation volume from an elegant bacterial system. Their chimeric VLP was based on the SARS-CoV-2 receptor binding domain that is fused to the cucumber mosaic virus. However, a direct comparison of the yield to our study is difficult because the molecular composition of this protein-rich non-enveloped particle is totally different [21].

In the future, it is necessary to use a uniform system to report the yield of VLPs to facilitate the comparison between production systems. We suggest a combination of nanoparticle tracking analysis of the unpurified cell culture supernatant and the purified sample. Furthermore, the total amount of purified protein per liter of cell culture as well as the concentration of specific protein (e.g., spike protein) of the purified sample would be beneficial. Such data would allow to compare the productivity of the cell culture system itself and the loss of product due to the applied purification method.

In a first proof-of-concept experiment, we show that the VLPs produced here are immunogenic. Mice that received two doses of VLPs had IgG antibodies against the nucleoprotein (50%) and the spike protein (100%). The serum neutralized lentiviruses pseudotyped with the SARS-CoV-2 spike protein. Of note, we have not yet tested the addition of adjuvants. Adjuvants drastically enhanced the immunogenicity of a plant-based VLP-vaccine candidate [23], which has now passed Phase III clinical trial [43] and received market authorization as COVIFENZ^®^ in Canada. Suitable adjuvants for the VLPs produced by our system as well as the final concentration of VLPs have to be determined in future experiments.

We also addressed the issue of inactivation of vaccine candidates over time. The stability of the compounds is important for the worldwide distribution of vaccines. Keeping a constant temperature can be particular challenging or sometimes even impossible in developing countries [44] if the product requires storage below freezing temperatures or even at ultra-low temperatures. In contrast to mRNA vaccines that need to be transported using dry ice, our VLPs are stable for at least 10 weeks at 4 °C. Encouragingly, similar or even longer shelf life has been reported for similar VLP-based vaccine candidates at 4 °C [26,30]. Naskalska et al. 2021 reported a similarly profound loss of particles when stored at −20 °C without cryoprotectants.

As a result of the continuous evolution of new SARS-CoV-2 variants of concern, a vaccine production system has to be adaptable. This essential feature was demonstrated by the rapid exchange of the spike protein in the VLPs produced in our system. In future experiments, it is important to explore the potential of the VLPs produced here and their variants to induce antibodies that neutralize current variants of concern.

## 5. Conclusions

In this study, we describe a stably modified mammalian SARS-CoV-2 VLP production system which minimizes economical and technical difficulties whilst achieving the highest particle yield reported so far in mammalian or insect culture systems. The produced VLPs are immunogenic and stable when stored at −80 °C or 4 °C.

## Figures and Tables

**Table 1:** Detection of all four structural proteins by mass spectrometry.

	MW [kDa]	# Amino Acids	# Detected Unique Peptides	Sequence Coverage (%)
S-protein	140.9	1273	80	58
N-protein	45.6	419	35	75
M-protein	25.2	223	6	26
E-protein	8.4	75	2	39

**Table 2:** Quantification of N- and S-proteins per VLP.

	Batch A	Batch B	Batch C	Mean	SD
*Cell culture*					
Culture volume (L)	0.38	0.40	0.11	0.30	0.13
Yield: VLPs/L culture	2.34 × 10^13^	2.25 × 10^13^	5.00 × 10^13^	3.20 × 10^13^	1.28 × 10^13^
Yield: mg protein/L culture	28.00	31.60	41.50	33.70	5.72
*Purified sample*					
Conc. VLPs/µL	1.78 × 10^9^	1.50 × 10^9^	2.26 × 10^9^	1.85 × 10^9^	3.14 × 10^9^
Conc. µg protein/µL	2.13	2.11	1.88	2.04	0.11
Conc. [N-protein] %	8.65	7.4	7.16	7.74	0.65
Conc. [N-protein] µg/µL	0.18	0.16	0.13	0.16	0.02
Conc. [S-protein] %	7.67	8.01	6.2	7.3	0.79
Conc. [S-protein] µg/µL	0.16	0.17	0.12	0.15	0.02
*Virus-like particle*					
N-protein(µg)/VLP	1.03 × 10^−10^	1.04 × 10^−10^	5.95 × 10^−10^	8.90 × 10^−10^	2.09 × 10^−10^
N-protein molecules/VLP	1364.4	1374.04	784.86	1174.43	275.5
S-protein(µg)/VLP	9.17 × 10^−11^	1.13 × 10^−11^	5.15 × 10^−11^	8.53 × 10^−11^	2.54 × 10^−11^
S-protein molecules/VLP	391.77	481.6	220.2	364.52	108.44
S-protein trimers/VLP	130.59	160.53	73.4	121.51	36.15

Predicted molecular weight of the N-protein (45.63 kDa or 7.58 × 10^−14^ µg/protein) and S-protein (140.95 kDa or 2.34 × 10^−14^ µg/protein).

## Data Availability

All related data and methods are presented in this paper. Additional inquiries should be addressed to the corresponding author.

## Supplementary Materials

The following supporting information can be downloaded at: https://www.mdpi.com/article/10.3390/vaccines11091469/s1, Figure S1: Example of dynamic light scattering data for SARS-CoV-2 VLPs at different stages during the purification process, Figure S2: Complete membrane of Western blot in Figure 3, Figure S3: SARS-CoV-2 VLPs are stable in solution at 4 °C and −80 °C for four weeks but not at −20 °C, +20 °C or +36 °C.
