# Supplementary material for: An Efficient and Scalable Method for the Production of Immunogenic SARS-CoV-2 Virus-like Particles (VLP) from a Mammalian Suspension Cell Line
Source: Vaccines (Basel). 2023 Sep 9;11(9):1469. doi: 10.3390/vaccines11091469 (PMC10535180; doi:10.3390/vaccines11091469)
Supplement: Supplementary file 1 [file vaccines-11-01469-s001.zip › vaccines-2559053-supplementary.pdf]

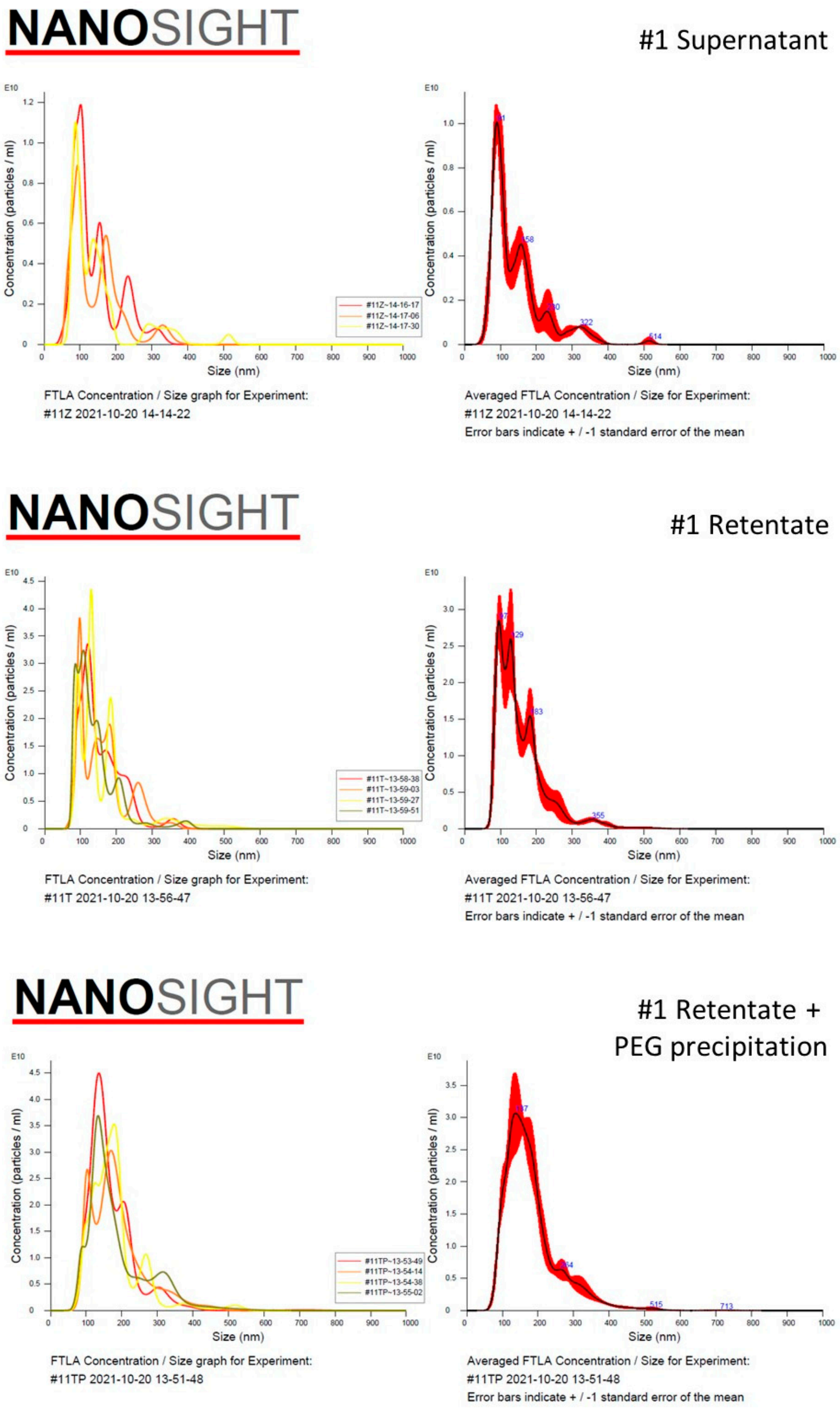

Figure S1: Example of dynamic light scattering data for SARS-CoV-2 VLPs at different stages during the purification process. Reference to Figure 3.

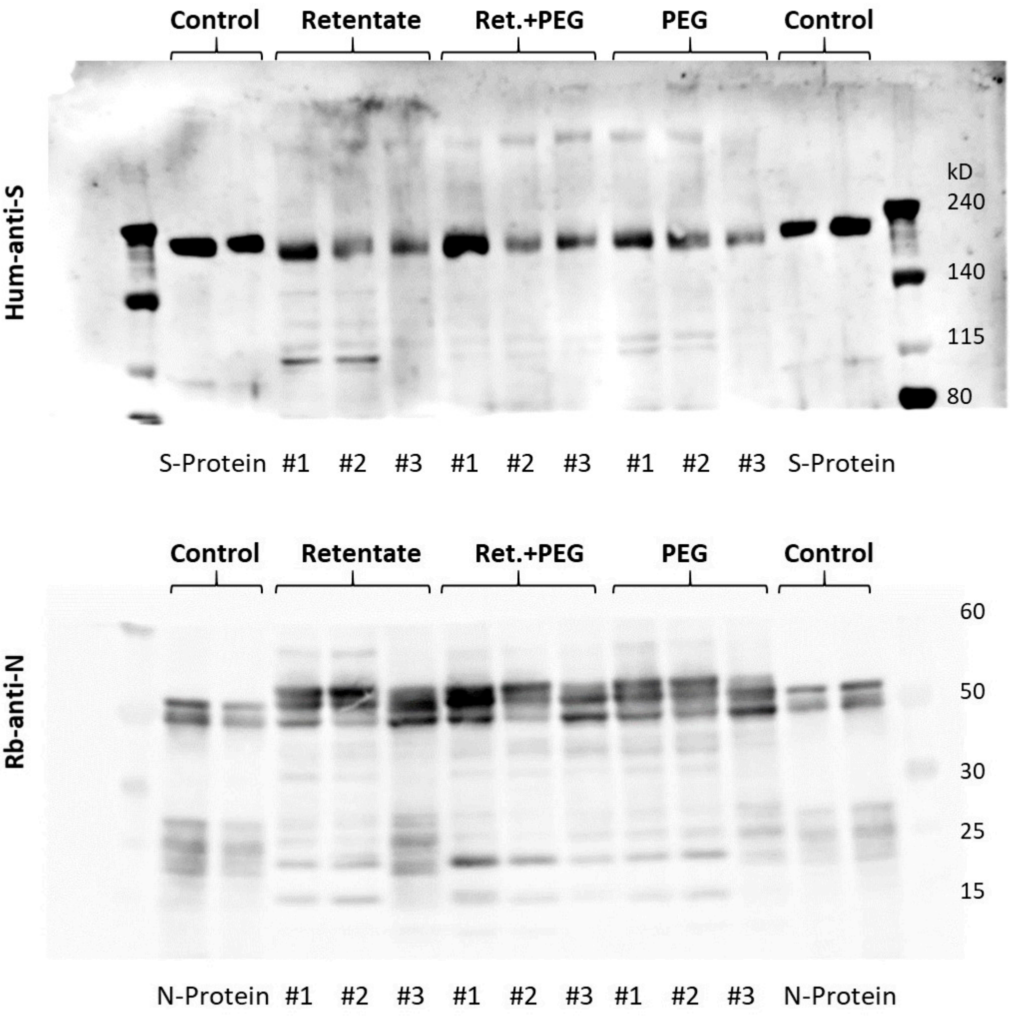

Figure S2: Complete membrane of Western blot in Figure 3

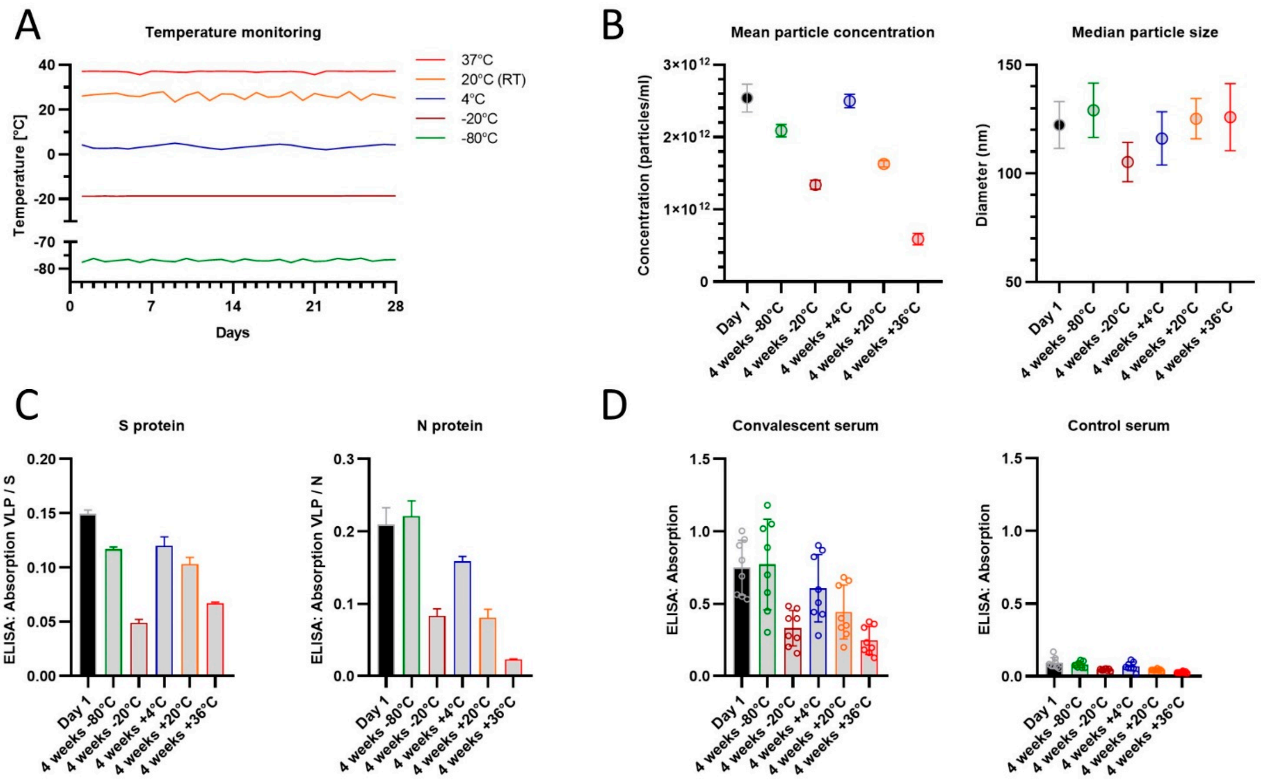

Figure S3: SARS-CoV-2 VLPs are stable in solution at 4°C and -80°C for four weeks but not at -20°C, +20°C or +36°C. (**A**) Storage temperature monitored by a data logger. The quality of VLPs was assessed after 4 weeks by (**B**) NTA, (**C**) spike- and nucleoprotein ELISA and (**D**) by the reactivity of the VLPs with human convalescent (n = 16) and control serum (n = 16).
